# The Development of Laparoscopy—A Historical Overview

**DOI:** 10.3389/fsurg.2021.799442

**Published:** 2021-12-15

**Authors:** Ibrahim Alkatout, Ulrich Mechler, Liselotte Mettler, Julian Pape, Nicolai Maass, Matthias Biebl, Georgios Gitas, Antonio Simone Laganà, Damaris Freytag

**Affiliations:** ^1^Department of Obstetrics and Gynecology, University Hospital of Schleswig-Holstein, Kiel, Germany; ^2^Institute of History and Ethics of Medicine, University Medical Center Hamburg-Eppendorf, Hamburg, Germany; ^3^Department of Surgery, Campus Charité Mitte and Campus Virchow-Klinikum, Charité - Universitätsmedizin Berlin, Berlin, Germany; ^4^Department of Obstetrics and Gynecology, Campus Charité Mitte, Charité - Universitätsmedizin Berlin, Berlin, Germany; ^5^Department of Obstetrics and Gynecology, “Filippo Del Ponte” Hospital, University of Insubria, Varese, Italy

**Keywords:** laparoscopy, history of medicine, Semm, kelling, minimally invasive surgery, Kiel

## Abstract

The advent of laparoscopy marked a fundamental change in the evolution of medicine. The procedure progressed consistently after the first time it was performed in a human being nearly a hundred years ago. The 1960's and 1980's witnessed groundbreaking changes. During this time, laparoscopy evolved from a purely diagnostic procedure into an independent surgical approach. Outstanding pioneers of the times were Palmer, Frangenheim and Semm. Laparoscopy advanced rapidly and influenced gynecology as well. The procedure was initially attacked most vociferously by the surgical fraternity. However, within a short period of time the pendulum shifted: laparoscopy became the preferred surgical approach for a variety of diseases—whether benign or malignant—in several medical disciplines. Laparoscopy has become a routine approach in the twenty-first century. Technical advancements have led to robot-assisted surgery. Future developments will include artificial intelligence and augmented reality. In the present article we address past milestones, current practices, and future challenges in laparoscopy.

## Introduction

*Primum non nocere*. Possibly, the Hippocratic impulse underlying the demand for moral action on the part of doctors was one of the many factors responsible for the introduction of minimally invasive surgery as we know it today. Surgeons and physicians have been consistently focused on minimizing the risks and complications of surgery.

Endoscopic procedures have become an integral part of all surgical specialties and are now a standard approach in all fields of surgery. An increasing number of complex surgical procedures are performed by the laparoscopic approach. The generation of the 2000s takes laparoscopy for granted. Conventional laparoscopy has been extended to include robotic-assisted surgery. In fact, we are on the verge of implementing artificial intelligence and augmented reality in laparoscopy (1–3). The history of laparoscopy reveals that a large number of steps and individuals were involved in the establishment of complex surgical techniques as we know them today (4–6).

Georg Kelling was the first to describe the basic principles of endoscopy of the abdomen. Kelling performed the procedure in a dog (5–8). Almost exactly a hundred years ago, Jacobaeus performed the first endoscopy in humans. Major advancements in endoscopy were accomplished from the 1960s to the 1980s, accompanied by a transition from diagnostic to surgical laparoscopy. These developments are inseparably linked with the names of Raoul Palmer in Paris and Kurt Semm in Kiel (5).

The first laparoscopic appendectomy was performed by Semm on 13 September 1980 at the department of obstetrics and gynecology, University of Kiel (5, 9, 10). It was an absolute rarity and an international sensation at the time. As a gynecologist and trained toolmaker, Semm revolutionized the course of traditional surgery. However, he aroused the criticism of many of his colleagues in gynecology and surgery. In his words, the medical world at the time reacted with the most violent hostility and opposition he had experienced during his entire career (9): “Both surgeons and gynecologists were angry with me, they virtually stoned me. All my initial attempts to publish a report on laparoscopic appendectomy were rejected with the comment that such non-sense does not, and will never, belong in general surgery.” Thus, his first report on laparoscopic appendectomy was published no earlier than 1983 (10). In an interview, his close colleague Liselotte Mettler (born in 1939) said that Semm was summoned from the operating room and had to undergo a computed tomography investigation of his skull in order to prove that he was in good health.

In a letter written in 1981, the President of the German Society of Surgery urged the German Society of Obstetrics and Gynecology to revoke Semm's medical license. The fact that a gynecologist wished to show surgeons how to perform an appendectomy was simply inconceivable. Semm had exceeded a limit that was considered impassable until the time. However, he was cognizant of the immense potential of laparoscopy not only in gynecology but also in surgery, and persisted in his unwavering efforts to minimize surgical trauma for patients (5, 9).

The rapidity of this development had a significant impact, especially on gynecology. Entirely new pathways could be broached after the introduction of laparoscopy in hysterectomy, uro-gynecological interventions, and oncological surgery, including lymphadenectomy in different compartments of the body. Laparoscopy became firmly established in other specialties as well, such as surgery and urology.

The increasing complexity of the interventions was accompanied by significant demands on surgeons and technological equipment. A surgeon comes close to his physical and mental limits when performing endoscopic operations for several hours (1).

The subsequent development of endoscopy was closely linked with technical advancements. The aim of laparoscopic interventions such as NOTES (natural orifice transluminal endoscopic surgery), or surgery performed through a single trocar (single-port technique) is to reduce access-related trauma by further minimization of the skin incision.

Robot-assisted surgery is the most dynamic form of minimally invasive surgery in our times. Better visualization of the field of surgery by means of 3D technology and extension of surgical instruments to 7 degrees of freedom permit the use of minimally invasive surgery even in complex situations. Robot-assisted guidance of the instruments enables the surgeon to work without tremor and with a low level of fatigue, which is very useful for surgeons as well as patients during complex interventions. Moreover, the surgeon is able to work simultaneously at two consoles. The learning curve is shortened, complication rates are reduced, and training in surgery is fostered (1).

The introduction of endoscopy in surgical practice is one of the greatest success stories in the history of medicine. As far as the development of minimally invasive options is concerned, there is still no end in sight.

This report provides a historical overview of laparoscopy from its early beginnings to current times.

## History and Pioneers

Endoscopy is derived from Greek and means “viewing the inner spaces of the human body” (“endo” and “skopein”). In addition to the usual investigation methods of palpation, auscultation and percussion, the very first written records of medicine bear evidence of the fact that doctors were always interested in the possibility of “looking into” (endoscopy) the human body (4).

Hippocrates II (born in 460 B.C., died in 375 B.C.), the principal representative of the school of Kos, described the use of a speculum for investigation of the rectum (4). Similar instruments for examination of the vagina were found in the ruins of Pompeii (destroyed in 70 A.D.) and have been described in other cultures as well. However, the scope of investigation was limited by the need to illuminate the field of investigation. Illumination has been a persistent issue in the history of laparoscopy (4).

Albukasim (912-1013 A.D), an Arabian physician, was the first to use reflected light to view the inside of the body. He held a glass mirror in front of the vulva and thus reflected light into the vaginal vault (4). Cardan (1501–1576) was the first to use a mechanical lamp. Aranzi (1530–1589), a Venetian, bundled light with the aid of a camera obscura. In 1768, the French gynecologist and surgeon George Arnaud de Rosil (1698–1774) constructed the first endoscopic investigation lamp with a shielded lantern. He was able to illuminate the vagina, previously unfolded with specula (11).

**Figure 1:**
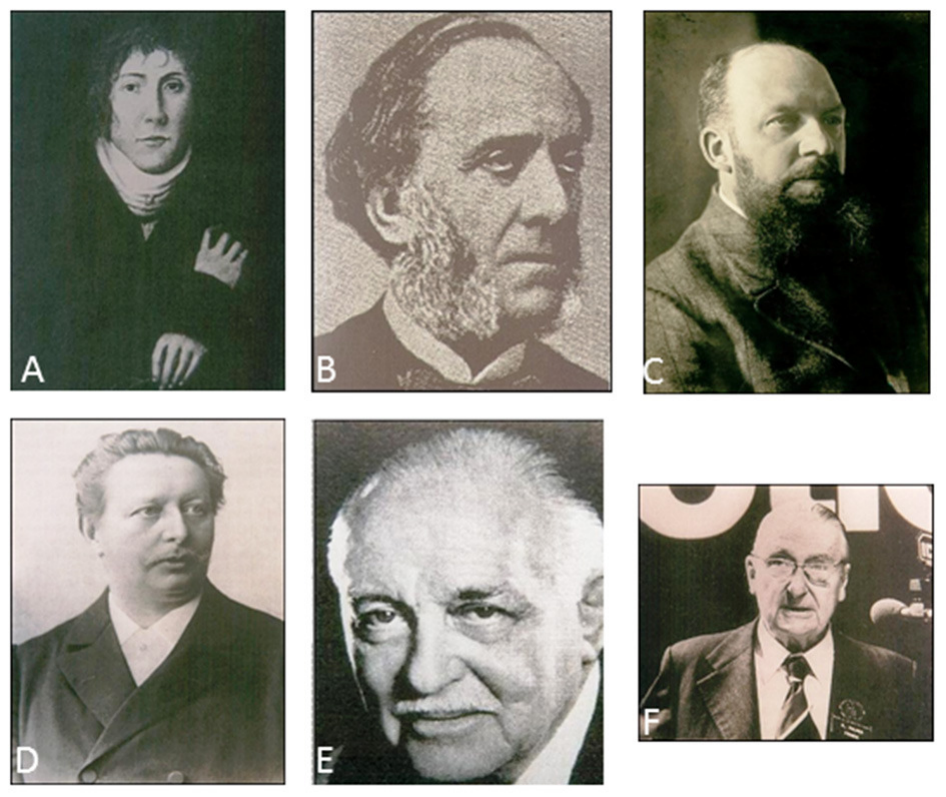
Pioneers of laparoscopy. **(A)** Philipp Bozzini (1773–1809), **(B)** Antonin Jean Desormeaux (1815–1894), **(C)** Georg Kelling (1866–1945), **(D)** Maximilian Nitze (1848–1906), **(E)** Heinrich Kalk (1895–1973), **(F)** Raoul Palmer (1904–1985).

Philipp Bozzini (1773–1809), a doctor in Frankfurt, played a significant role in the development of modern endoscopy because he marks the transition from ancient to modern medicine (12). In 1806 Bozzini (Figure 1) published his report about his light conductor, a device consisting of an optical part with lighting equipment and a mechanical part aligned to the anatomy of the body orifice (12). He thus created an instrument for the vagina, the rectum, and the oral cavity, which could be used for inspection and to a lesser extent for performing surgery. Although the light conductor was much too weak and the field of vision too small, all further attempts to perform cystoscopy during the next 70 years were based on Bozzini's principle of illumination, namely that of a reflected extracorporeal light source (5, 12).

Antonin Jean Desormeaux (1815–1894) advanced this historical development in 1843 with the creation of the first portable endoscope (13). Antonin Jean Desormeaux was the first to clinically use Bozzini's light conductor for which many regard him as the “father of endoscopy” (Figure 1). His endoscope was a system of mirrors and lenses with an open flame as the light source, and was primarily used for the clarification of urological questions. One of the disadvantages was the enormous heat generated by the light source, which led to burns (13). Endoscopes illuminated by electricity were developed only after the invention of the light bulb by Thomas Alva Edison in 1879, and the miniature version of the bulb in the form of a mignon lamp (5). Subsequent developments in endoscopy were initially focused on cystoscopy. Maximilian Nitze (1848–1906) modified Edison‘s light bulb. In 1877 Nitze (Figure 1) created the first urethroscope and cystoscope with an integrated light source for the illumination of body cavities, and thus laid the foundations of clinical endoscopy (14). Johann Mikulicz-Radecki (1850–1905) and Joseph Leiter, an instrument maker from Vienna, adopted Nitze's principle of a rigid optical system in 1881 and developed the first gastroscope for clinical use (15, 16).

### Era of Diagnostic Laparoscopy (1901–1933)

In 1901 Georg Kelling (1866–1945), a surgeon and gastroenterologist from Dresden, demonstrated the first laparoscopy at the end of his lecture entitled “About the inspection of the esophagus and the stomach with flexible instruments”; he referred to the technique as “coelioscopy” (6). In his doctoral thesis, Kelling had focused on the anatomy and physiology of the gastrointestinal tract. Based on this knowledge and his discoveries about the insufflation of air into the abdomen, he was the first to develop the method further. He investigated the abdominal cavity of a dog he had insufflated earlier with filtered air, using a Nitze cystoscope. This invervention may be regarded as the natal hour of laparoscopy (7, 8). At the time, Kelling (Figure 1) concluded his lecture with the following words (6): “Gentlemen, I conclude this lecture by expressing the hope that endoscopic methods will be used to a greater extent in the digestive tract than has been common practice so far. In fact, in many instances, endoscopic methods are destined to replace laparotomy.” However, Kelling is not known to many as the primary creator of the technique. One of the reasons is definitely the course of Kelling's life. Along with his second wife, he died in a heavy air attack on Dresden in February 1945. His reports and personal documents were lost at the time (6–8, 17).

Nine years later, the Swedish internist Hans-Christian Jacobaeus (1879–1937) introduced the term “laparothoracoscopy” during the first endoscopic inspection of the human chest and abdominal cavity (18, 19). Jacobaeus published his experience of the first 17 laparoscopies in 1910 in the “Münchner Medizinischen Wochenschrift” (Munich Medical Weekly). The report was entitled “About the options of using cystoscopy for the investigation of serous cavities” (19). Jacobaeus recommended the technique for the endoscopic inspection of other body cavities as well. In contrast to Kelling, Jacobaeus introduced the trocars directly without creating a pneumoperitoneum. Jacobaeus started to release adhesions by viewing the body with the aid of thoracoscopy. He performed thoracoscopic investigations as early as 1913 and is therefore regarded as the founder of thoracoscopic operations (18, 19).

The first laparoscopy in the USA was performed by Bertram M. Bernheim (1880–1958) in 1911. He named his method organoscopy (20, 21). The instruments consisted of a proctoscope and simple lighting. Bernheim introduced his instrument through a mini-incision without creating a pneumoperitoneum (20). He was able to detect an enlarged gall bladder in his first patient. However, the actual diagnosis of a pancreatic carcinoma was established only after a subsequent laparotomy. After publication of his organoscopy, Bernheim lost interest in gastroenterology and devoted his attention to vascular surgery (21).

Insufflation of the abdominal cavity was improved. One day after the end of the First World War, a treatise on X-ray diagnosis of the abdominal cavity was published in the Munich Medical Weekly. Otto Goetze (1886–1957), an assistant surgeon and author, focused on radiological problems. He used oxygen to improve contrast on X-rays (22). In order to introduce oxygen safely into the abdomen, he developed a double-walled cannula in accordance with the “principle of solid displacement.” The term “pneumoperitoneum” was also coined by him (22). In 1924 the Swiss gynecologist Richard Zollikofer replaced air with CO2 for the purpose of insufflation (19).

Laparoscopy became increasingly well-known and its application spread far and wide. The existing knowledge had to be summarized in a textbook. Roger Korbsch published the first textbook on the subject in 1927 (23).

Optics were also improved. Heinz Kalk (1895–1973), a gastroenterologist from Berlin, known as the founder of the German school of laparoscopy, developed a 135-degree lens system and a double trocar (24). Kalk (Figure 1) used laparoscopy as a diagnostic procedure for diseases of the gall bladder and the liver. In a publication of his experiences in 1939, he reported on more than 2,000 liver punctures under local anesthesia without encountering a single fatality. He also released adhesions by the laparoscopic procedure (5, 24).

### Era of Operative Laparoscopy

Apart from significant progress in equipment and technology, the surgical spectrum was widened as experience with the technique increased. In 1933, Carl Fervers succeeded in performing the first laparoscopic adhesiolysis, which may be regarded as the first surgical laparoscopy in the current sense of the term (25).

In 1937, the American J.C. Ruddock reported on more than 500 laparoscopic procedures with biopsies, mainly obtained from the liver. This internist, who published his work in surgical journals, used pincers supplied with electrical power for the purpose of coagulation. Notably, Ruddock was the first to report a substantial number of complications with the technique (26).

A further milestone in the historical development of laparoscopy was achieved by the Hungarian internist and pulmonologist János Veres (1903–1979), who introduced his insufflation needle which had, in fact, been described earlier by Goetze but had slipped into oblivion. Veres developed a special canula with a spring mechanism. Its purpose was to create a pneumothorax in order to treat tuberculosis, which was a widespread disease at the time (27). Even today, the Veres needle is used to create a pneumoperitoneum safely in laparoscopy. Due to its spring mechanism, it permits gas insufflation with a low rate of complications and prevents injury to internal organs when being introduced through the abdominal wall (27).

The first minor surgical interventions were performed in the sixties of the twentieth century, primarily by gynecologists. Raoul Palmer (1904–1985), a gynecologist from Paris, made notable achievements in this regard (28). Palmer (Figure 1) was mainly concerned with the diagnosis of sterility and its treatment. In 1944 he performed a laparoscopy in Trendelenburg position. He also conducted the first sterilization by laparoscopy. Although an incision site in the upper abdomen in the caudal aspect of the left costal margin is named after Palmer, in 1946 he performed the incision in the navel and preferred this site. Like Kelling, he also referred to the endoscopic procedure as coelioscopy (28).

Since the abdominal access was difficult because of the blind nature of insertion, in 1946 the American Albert Decker (1895–1988) introduced the laparoscope transvaginally through the posterior vaginal vault and named the procedure culdoscopy. However, the diagnostic procedure was inadequate from this perspective and the technique, which was initially rather widely accepted, became less popular in the USA (29).

### Pioneers of Operative Endoscopic Surgery

**Figure 2:**
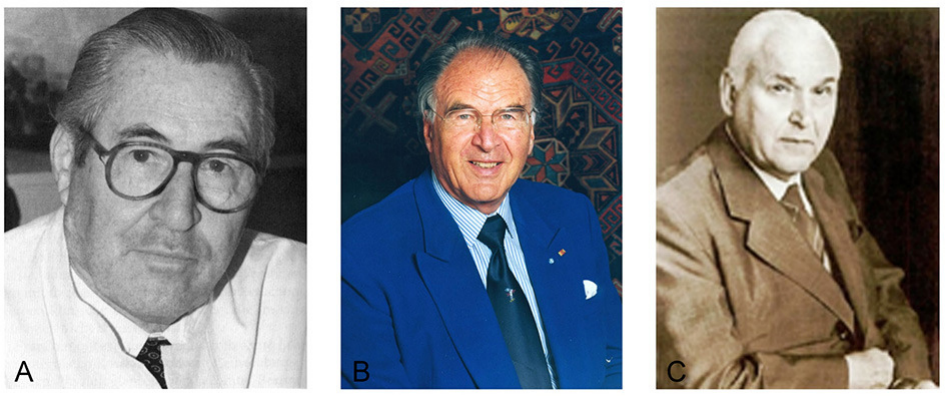
Pioneers of operative laparoscopy. **(A)** Hans Frangenheim (1920–2001), **(B)** Kurt Semm (1927–2003), **(C)** Karl Storz (1911–1996).

The development of laparoscopy in Germany after the Second World War is primarily linked with the names of Hans Frangenheim (1920–2001) and Kurt Semm (1927–2003) (Figure 2). Frangenheim encountered laparoscopy in 1952, when a tumor in the lower abdomen was discovered during an endoscopy of the liver at the Medical Clinic of Cologne, and the subsequent strategy had to be decided. In 1955 he attended a clerkship under Palmer in France and realized that laparoscopy was superior to culdoscopy, which was being used in Germany at the time. He devoted his efforts to the development of new instruments, photographic documentation of endoscopic reports, and the improvement of gas insufflation which was completely uncontrolled until this time. In cooperation with Dräger Company, Frangenheim developed a CO2 insufflator. His monographs, publications and lectures contributed to further dissemination of the method (30, 31).

The Kiel University Department of Obstetrics and Gynecology under Kurt Semm (1927–2003) is regarded as the birthplace of modern laparoscopy. He became the most productive researcher and innovative instrument maker in the field of modern endoscopic surgery (18).

**Figure 3:**
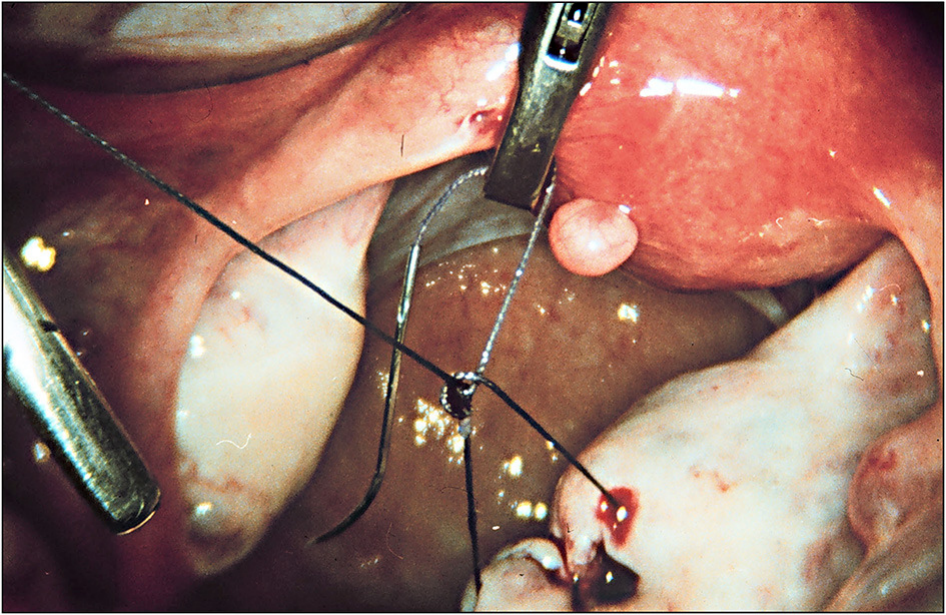
Introduction of intracorporeal knots (1974). Source: Department of Obstetrics and Gynecology, University Clinic of Kiel.

In the 1960s, two decisive developments paved the way for a new era in endoscopy. The British physicist Harold Hopkins (1918–1994) developed the Hopkins optics in 1961 and achieved a further milestone in the field (32). This optical instrument consisted of so-called rod lenses. Its eighty-fold higher light transmission and enlarged field of vision yielded sharper and brighter images. The technique attracted the attention of the German instrument maker Karl Storz (Figure 2), who convinced Hopkins to work with him. As early as 1960, Karl Storz Company developed the cold light source, which replaced the bulb at the tip of the endoscope. The advantages of the cold light source were obvious: it provided much better illumination and caused less heat (32). Until 1970, intra-abdominal endoscopy was largely confined to diagnostic procedures. This limitation could be attributed to the fact that there were no means of arresting bleeding after surgical interventions. Semm aimed to use laparoscopy for purposes other than diagnosis alone. In order to distinguish this technique from internistic laparoscopy of the upper abdomen, he named his procedure pelviscopy (4). Being a precision mechanic himself, Semm developed many instruments personally. He had been engaged in endoscopy since 1955. His automatic CO2 insufflator, constructed in 1963, made operations in the abdominal cavity safer and more comfortable. Thermocoagulation was introduced in 1973, followed by the Roeder loop to stop bleeding (33). Semm developed a special suction irrigation device, an electronic insufflator, and the first morcellator in 1977. His spectrum of instruments and methods of hemostasis (endosuture with intra- and extracorporeal knots, as shown in Figure 3) enabled surgeons to perform increasingly complex surgical procedures (5). These innovations proved to be crucial prerequisites for the subsequent development of endoscopy.

However, a large number of gynecologists and surgeons criticized Semm for his “keyhole surgery” and believed that, thanks to modern anesthetic methods, laparotomy was no longer a problem. According to these critics, Semm had exaggerated the problem of adhesions which, they believed, could be easily resolved by open laparotomy. Some physicians treated Semm's reports of the new spectrum of endoscopic options (treatment of tubal pregnancy, adnexectomy, or ovariectomy) with disbelief, and asserted that Semm started an operation as a laparoscopy and ended with a laparotomy (5). Semm met with fierce resistance when he performed the first laparoscopic appendectomy in 1980, because surgeons saw no reason to replace an established surgical method by a technically complicated one (9, 10). The circumstance of a gynecologist showing surgeons how to perform an operation was simply inconceivable. Semm had crossed a limit that was considered impassable until the time. Cognizant of the immense potential of endoscopic surgery not only in gynecology but especially in surgery, Semm pursued his work in laparoscopy with unswerving commitment. He persisted in his efforts to reduce surgical trauma for patients (5, 9).

Two German surgeons, Gotz and Pier, pursued Semm‘s purpose and established laparoscopic appendectomy on a wide basis. Already in the early 90's, they performed hundreds of appendectomies by this approach and perfected the technique. They even used it in patients with acute appendicitis (5).

In 1985, the German surgeon Erich Mühe (1938–2005) performed the first laparoscopic cholecystectomy using the instruments developed by Semm (34). In 1987, he reported on 97 successful operations performed by this technique (35). Reich et al. described the first laparoscopic-assisted hysterectomy in 1989, while Mouret performed the first cholecystectomy by video laparoscopy in 1991 (36). At the time, the industry realized the importance and potential commercial benefits of this development and became more interested in laparoscopy.

However, there was still vehement opposition. Hans Troidl, then senior surgeon at the University Clinic of Surgery in Kiel reported that, at the 107th Congress of the German Society of Surgery in Berlin in 1990, an article on the first 100 laparoscopic cholecystectomies initially submitted and published by him was suddenly struck off from the official Congress program (from a personal communication with the authors IA and UM).

A video presentation of Semm's laparoscopic appendectomy at a gynecologists' convention in Baltimore in 1988 encouraged McKernan and Saye to perform the first laparoscopic cholecystectomy in the USA. They used Semm's instruments in combination with laser technology (5). Later on, many endoscopists visited the two surgeons in Nashville in order to learn the new technique. The news reached the media in the USA, and the technique was publicized in a talk show on television. This was followed by numerous training courses, which were fully booked within a very short period of time (5).

**Figure 4:**
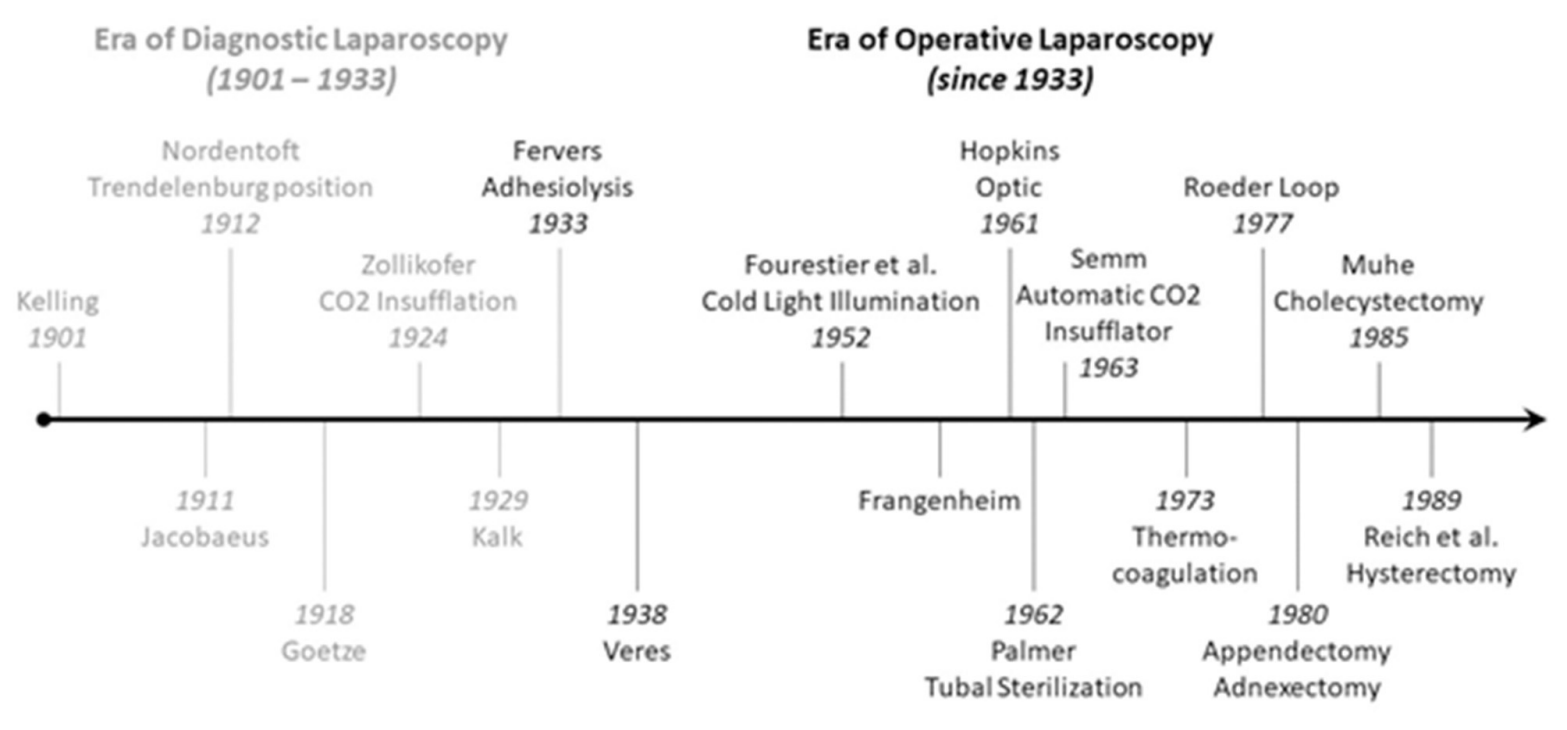
Timeline of the development of laparoscopy.

Figure 4 summarizes the historical steps of development from diagnostic to operative laparoscopy.

Advances in other fields of medicine delayed the subsequent evolution and application of laparoscopy. The development of laparoscopy was ignored by many surgeons in the 1970's and 1980's. The reasons were innovations in anesthesia and intensive medicine, and the new spectrum of medications which enabled surgeons to perform prolonged operations. Endoscopic cholecystectomy and appendectomy were considered daredevil artist's surgeries that offered risky solutions to safely resolved problems. The notion that major medical problems call for major solutions such as abdominal incisions was deeply ingrained in the minds of surgeons. The fundamental concept of laparoscopy was contrary to this notion (5).

### Announcement of “Minimally Invasive Surgery”

In contrast to general trends at the time, a group of German surgeons founded the Surgical Task Group for Endoscopy and Ultrasonography in 1976. Five years later, the Society of American Gastrointestinal Endoscopic Surgeons (SAGES) was founded in the USA.

The first issue of the journal Surgical Endoscopy was published in 1987 and the first World Congress of Surgical Endoscopy was held in 1988. The convention was a major success and is regarded as a global breakthrough in laparoscopy (5).

The British urologist John E.A. Wickham (born in 1927) was the first to use the term “minimally invasive surgery” and attracted significant attention when he published his visions about endoscopic procedures in 1987 in the British Journal of Urology (37). Although Wickham was exposed to substantial criticism, his ideas are reflected in the trend of the 1980's, when doctors and patients alike became fascinated with laparoscopic techniques (5).

In 1987 he predicted the paradigm shift in practical surgery that took place a little later: “Surgeons applaud large incisions and denigrate “keyhole surgery.” Patients, in contrast, want the smallest wound possible, and we at Britain's first department of minimally invasive surgery are convinced that patients are right” (37).

In the early 1990s, literally overnight endoscopic surgery was welcomed by widespread acceptance and rapid dissemination. This “laparoscopic revolution” was triggered by a sudden demand from patients, and its popularity heightened by avid media interest (38). The change was dramatic especially in cholecystectomy. The unprecedented demand gave rise to new problems: countless surgeons were unfamiliar with the new technique and had to undergo endoscopic training in a short period of time (38, 39).

**Figure 5:**
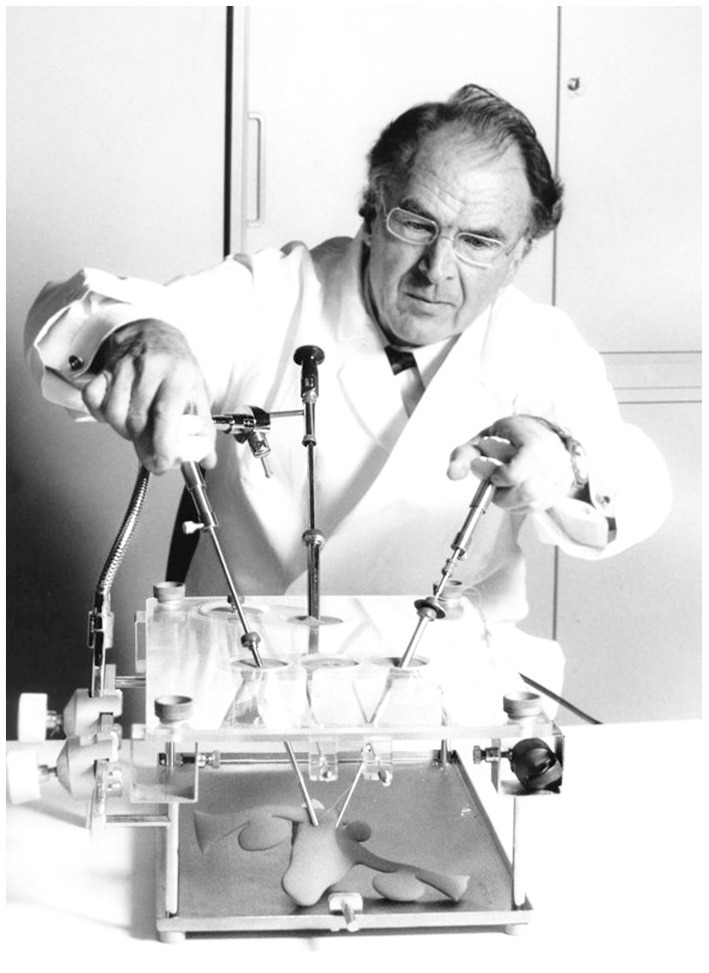
Pelvi trainer (1985). Source: Department of Obstetrics and Gynecology, University Clinic of Kiel.

The problem of imparting the knowledge and skills of endoscopy had been addressed for a long time. A breakthrough was achieved by the so called pelvi trainer (Figure 5), developed by Semm in 1985, which became an indispensable tool for learning laparoscopic techniques (40).

### Technical Breakthrough With Electronic Video-Endoscopy

**Figure 6:**
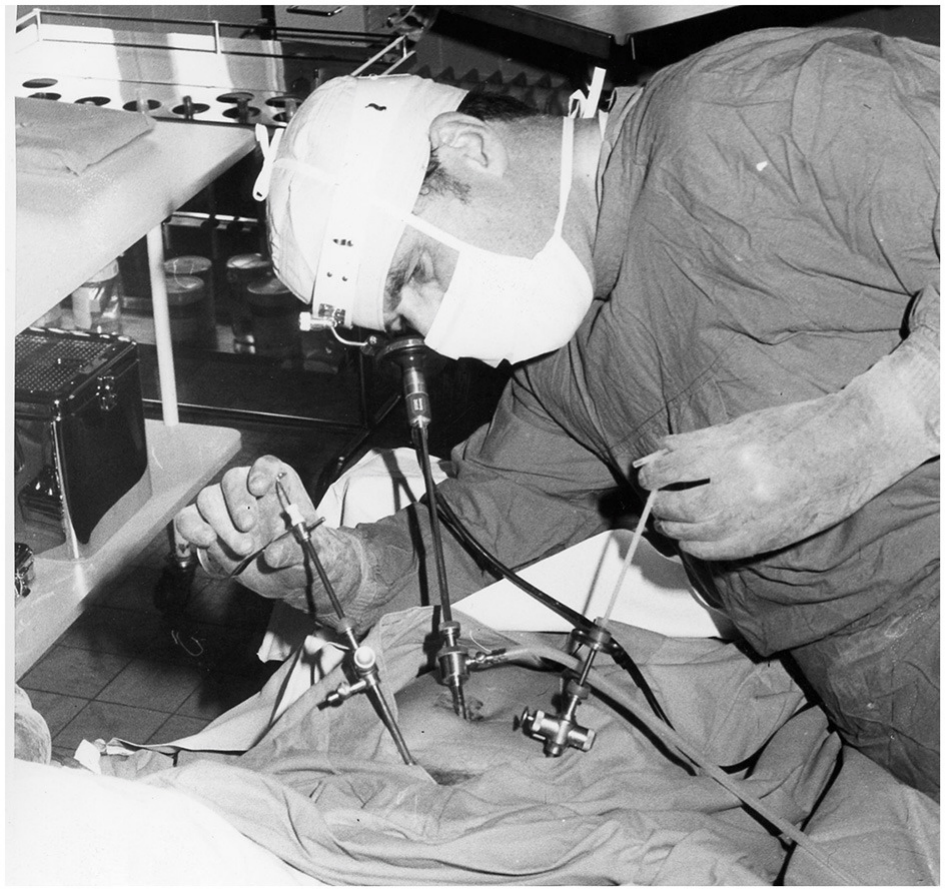
Around 1970 Semm developed the “head ring,” a simple device by which he freed his left hand. The optical device need not be held; it hangs on a small hook. Source: Department of Obstetrics and Gynecology, University of Kiel.

**Figure 7:**
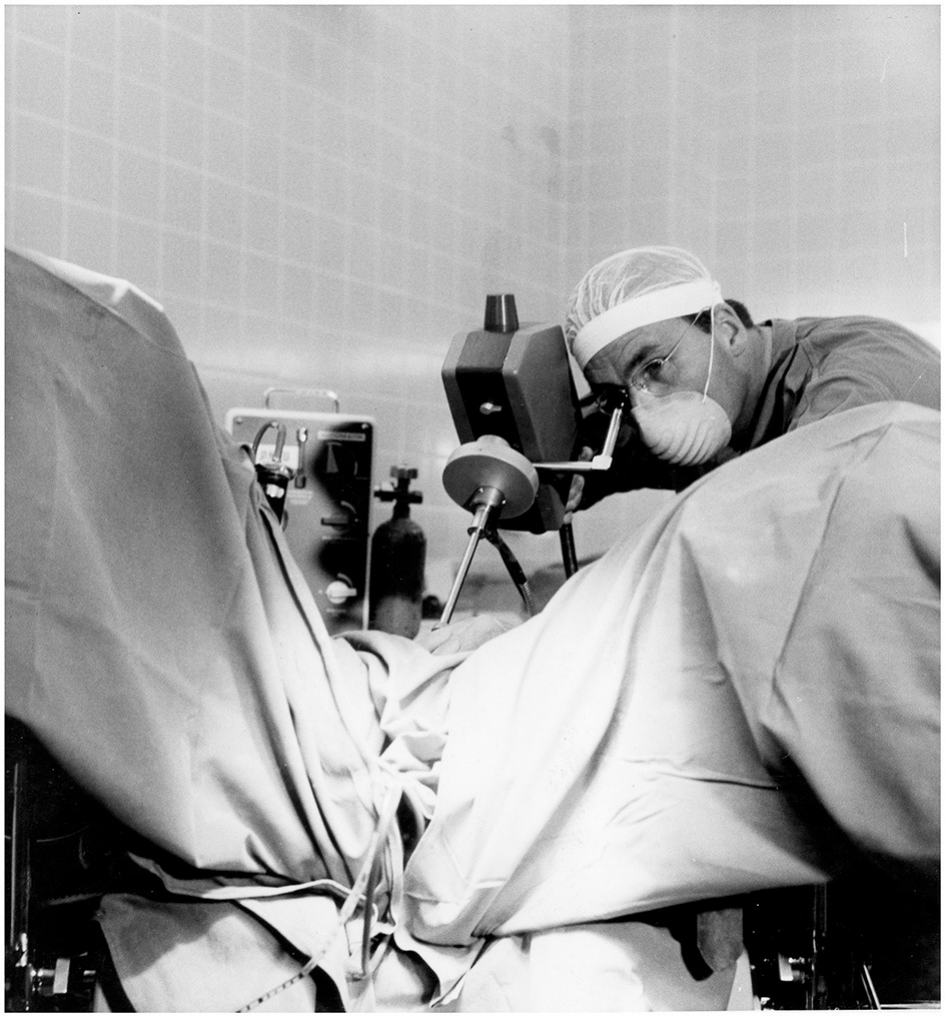
Always with the finger on the pulse of the latest technology. As early as 1970, just a few months after the German tech company Philips launched their first “portable” TV camera, Semm used it in the operating room. Several educational films were produced with the aid of the camera (41–43).

**Figure 8:**
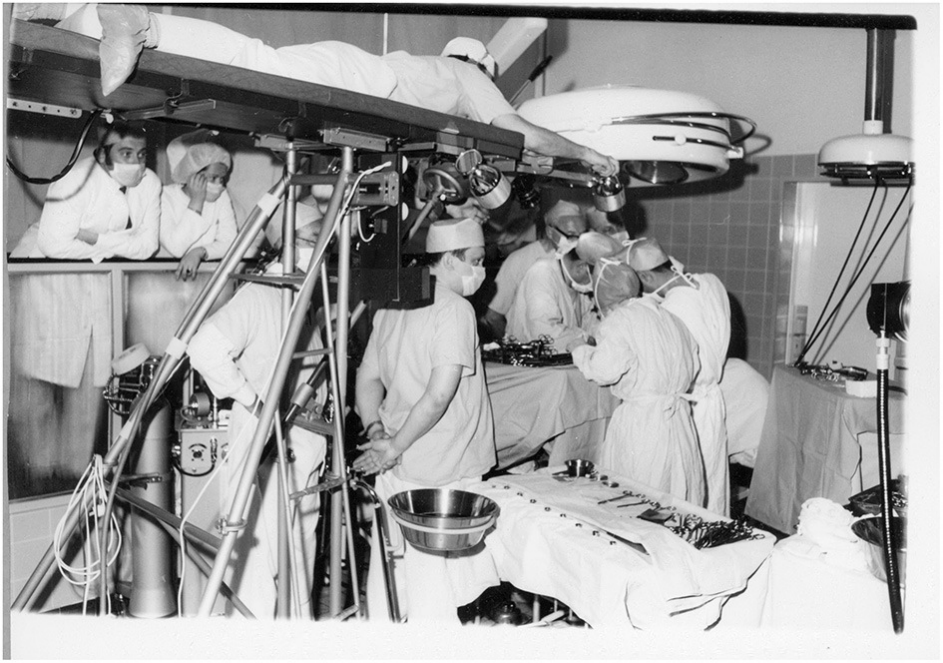
Technical breakthrough: electronic elements in the operating room at the Wertheim week in Kiel, organized by Semm in 1972. The operation was performed by Soichi Sakamoto and Semm and demonstrated by camera broadcast to a large number of national and international guests (camera work by Volker Rimkus, Semm's assistant). Source: Original interview with Volker Rimkus (born in 1939).

**Figure 9:**
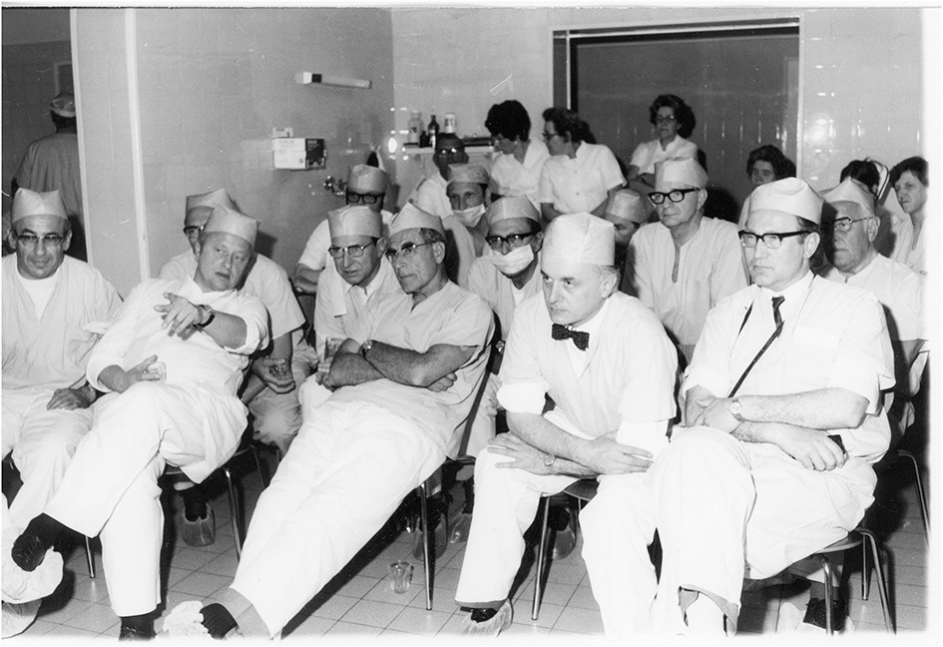
1972 Department of Obstetrics and Gynecology at the University Clinic of Kiel. The new technical options offered by the video camera enabled Semm to demonstrate his operations to a large number of national and international visiting doctors, who followed the operations with great interest. Semm referred to the event as the birth of video pelviscopy [33]. Source: Department of Obstetrics and Gynecology, University Clinic of Kiel.

**Figure 10:**
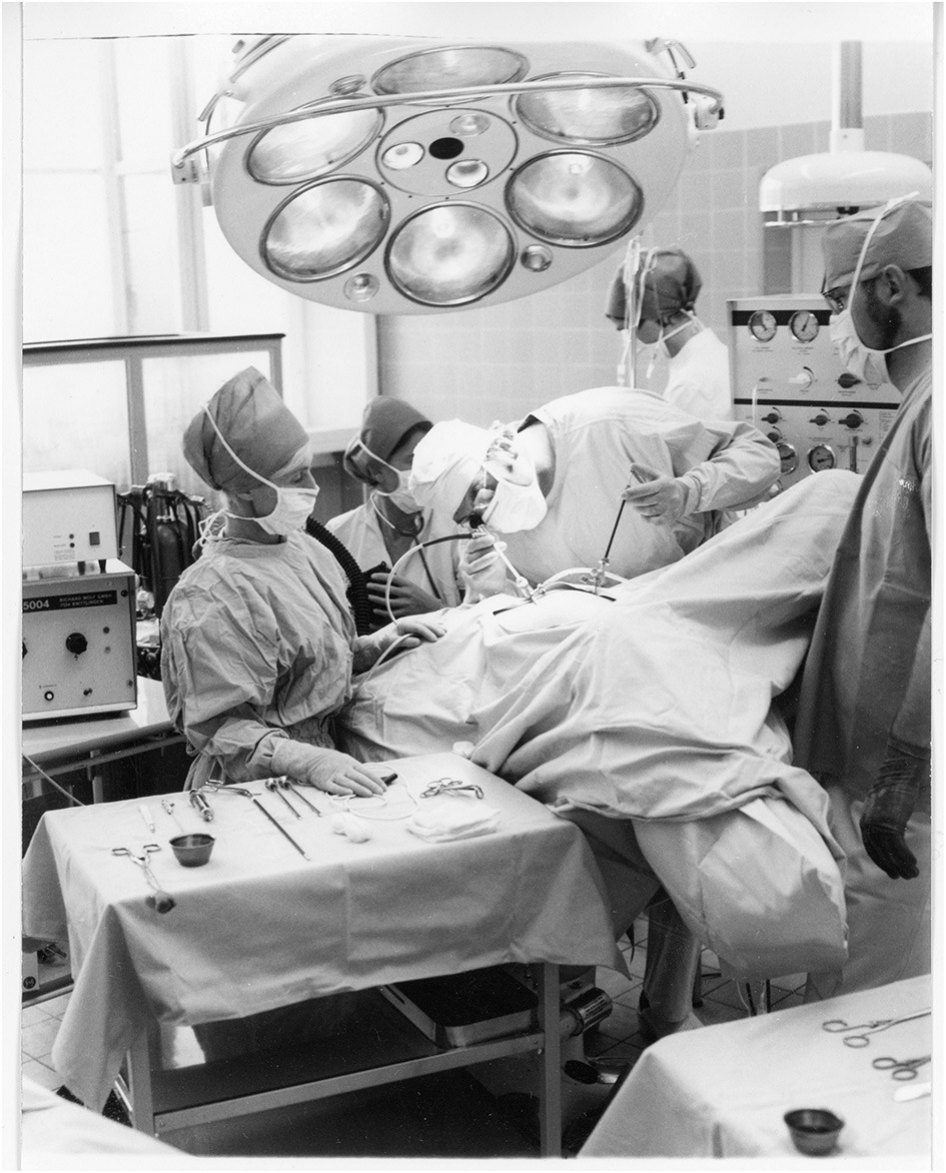
Semm performing a sterilization by laparoscopy. Source: Department of Obstetrics and Gynecology, University of Kiel.

The introduction of electronic elements in the operating room (as shown in Figures 6–10), along with video transmission, aided the breakthrough in operative endoscopy (41–43).

The traditional monocular endoscope does not permit the surgeon to share his vision with others. For a long time, the surgeon guided the optical instrument himself and had only one hand for surgical work (Figures 6, 10). The assistant surgeon had no means of viewing the field of surgery (as shown in Figure 10).

The introduction of the endoscopic video camera and image transmission to a monitor transformed the situation. The surgeon could now work with both hands and the assistant could follow the operation on the monitor. They could now operate as a team and demonstrate technical options to every observer. However, the first video cameras were heavy, cumbersome, and unsuitable for routine use. Electronic mini-cameras, introduced in 1987, resolved the problem (5).

### Instruments and Equipment for Laparoscopy—Past and Present

Palpators were the sole instruments used in endoscopy until 1960. The diagnosis and treatment of female sterility, and later tubal sterilization, were the main procedures performed in gynecological laparoscopy until 1970 (5). Therefore, the first instruments to be developed were scissors and atraumatic grasping forceps for transection of the fallopian tubes. Thermal methods of hemostasis were needed after 1970. The methods of hemostasis developed by Semm (thermocoagulation, Roeder loop, endosuture with intra- and extracorporeal knots) and his extensive range of instruments enabled surgeons to perform increasingly complex operations (5).

**Figure 11:**
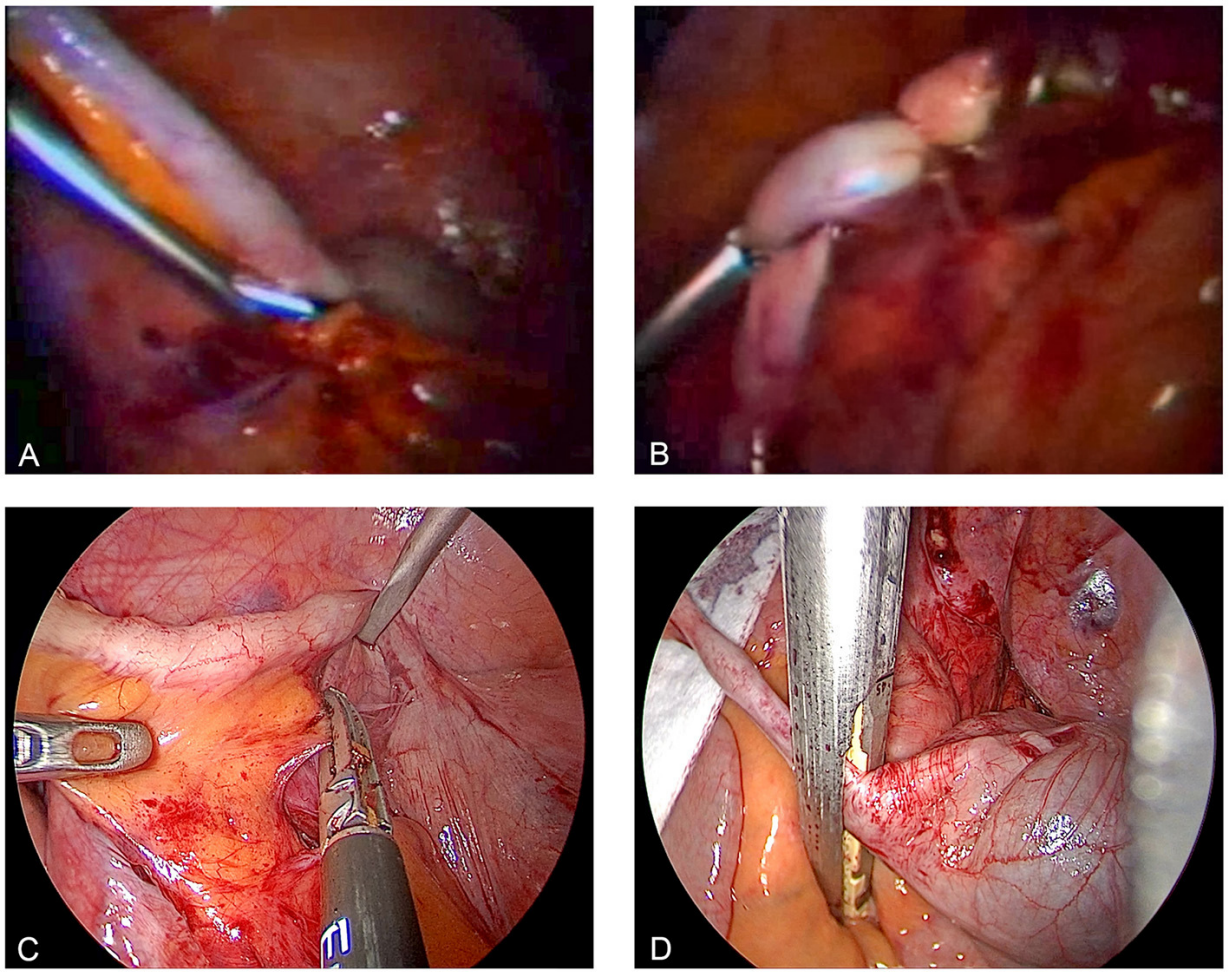
Laparoscopic appendectomy past and present. **(A,B)** are original pictures of the first laparoscopic appendectomy performed by Semm in 1980: skeletization of the appendix was followed by its ligation at the base with a Roeder loop. **(C,D)** show the current standard of surgery with the use of a stapler. The significantly better image quality and advanced instruments are evident. Source: Department of Obstetrics and Gynecology, University of Kiel.

**Figure 12:**
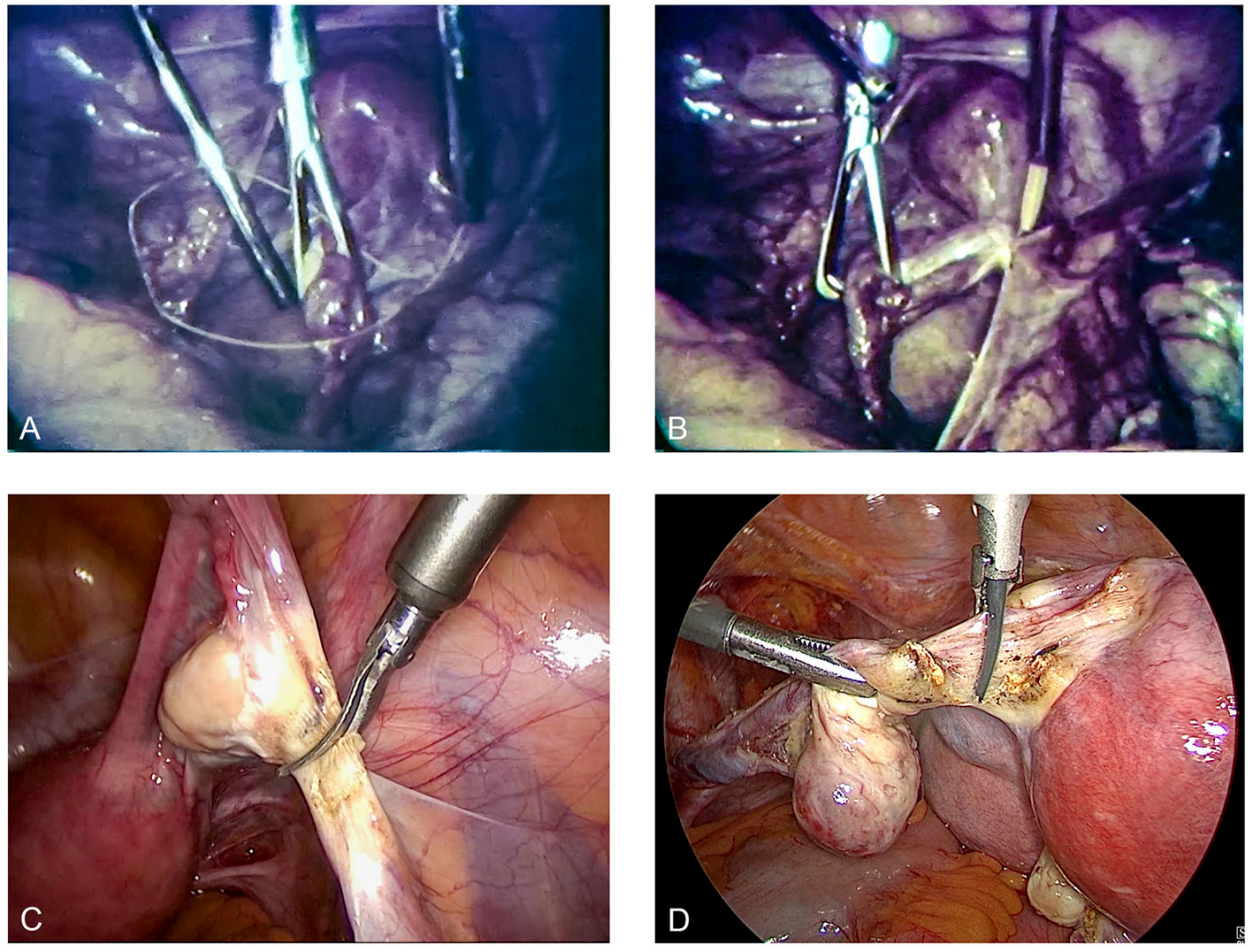
Laparoscopic adnectomy past and present. **(A,B)** are original pictures of a laparoscopic adnectomy performed by Semm using the three-loop method in 1980. **(C,D)** show the current standard of surgery. The markedly better image quality is evident. Source: Department of Obstetrics and Gynecology, University of Kiel.

**Figure 13:**
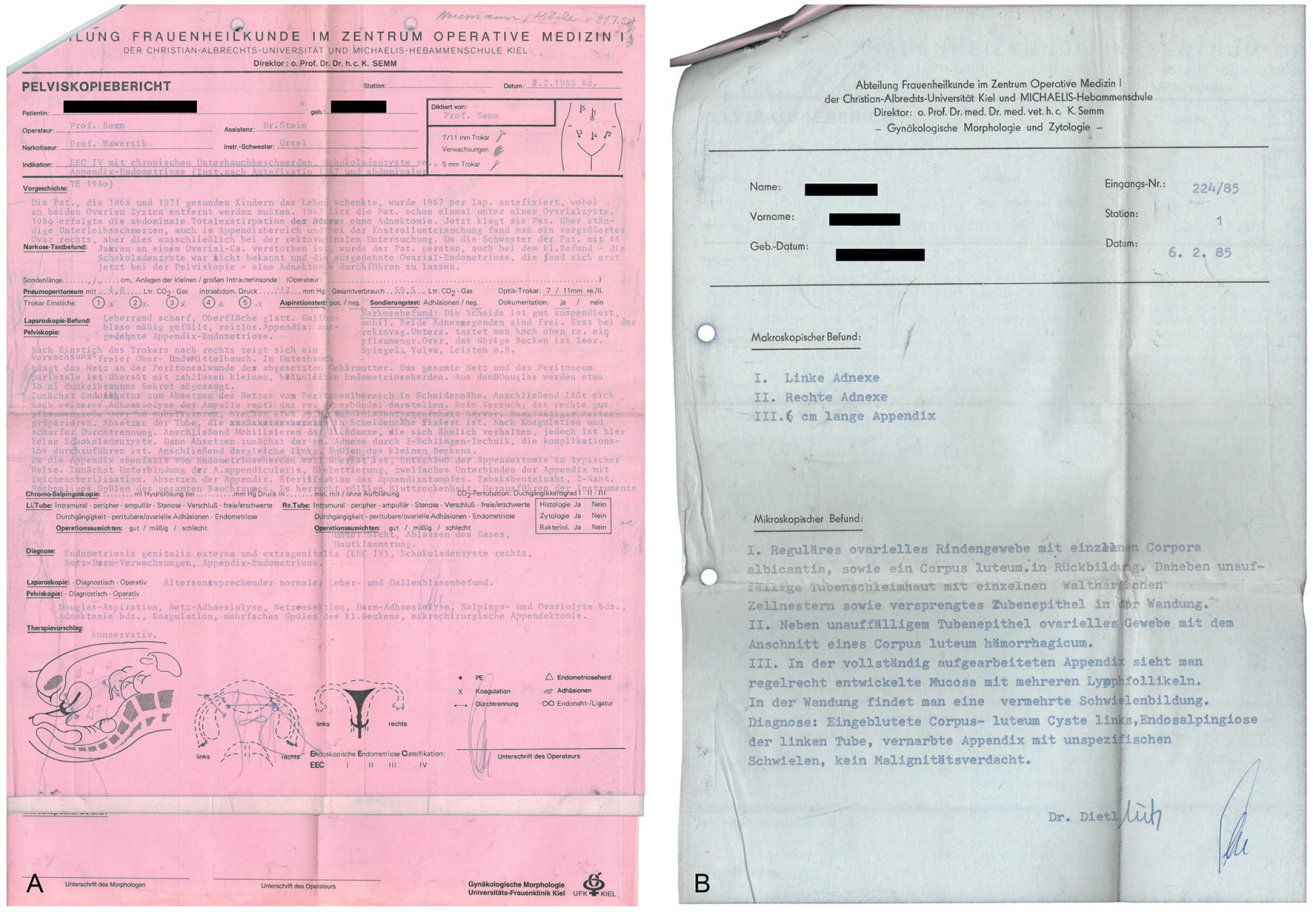
Original operation report by Semm in 1985. **(A)** Pelviscopy report of a patient who had undergone a hysterectomy, had chronic pain in the lower abdomen, suspected endometriosis of the external genitalia and extragenital endometriosis (endometriosis of the right-sided ovary, suspected endometriosis of the appendix). The patient underwent adhesiolysis, adnectomy on both sides by the 3-loop technique, and appendectomy. **(B)** Histological report showing evidence of a hemorrhagic corpus luteum cyst on the left side, endosalpingiosis of the left fallopian tube, and a scarred appendix. Source: Department of Obstetrics and Gynecology, University of Kiel.

Semm, who had completed an apprenticeship as a precision mechanic before entering medical school, found an effective means of putting his discoveries into clinical practice through his family. His father and brother were owners of WISAP, a company that produced medical instruments (9). While others had to wait for years before they could implement their ideas, Semm designs of new devices could be introduced in clinical practice within a short period of time (9). This aroused the envy of many (9). Semm's numerous innovations extended the spectrum of surgical options to a significant extent (such as laparoscopic management of ectopic pregnancies, tubal sterilization by endocoagulation, salpingostomy, oophorectomy, salpingolysis, fimbriolysis, lysis of omental adhesions, bowel suturing, repair of uterine perforations, and myomenucleation) (9). In 1980, Semm and his staff member Liselotte Mettler reported the first endoscopic ovariectomies and adnectomies performed with the aid of the Roeder loop. The same year, Semm performed the first laparoscopic appendectomy which he published in 1983 (5, 9, 10). Figures 11–13 show original pictures of the first appendectomy and adnectomy performed by Semm in 1980, and original operation reports of the procedures (10, 41). In contrast, the procedures as they are performed today. Technological advancements such as miniaturized video cameras, innovative instruments, HD image quality and 3D technology show where we stand 40 years later. These innovations were driven forward at a rapid pace to the present day by several pioneers of operative laparoscopy and the technical industry.

**Figure 14:**
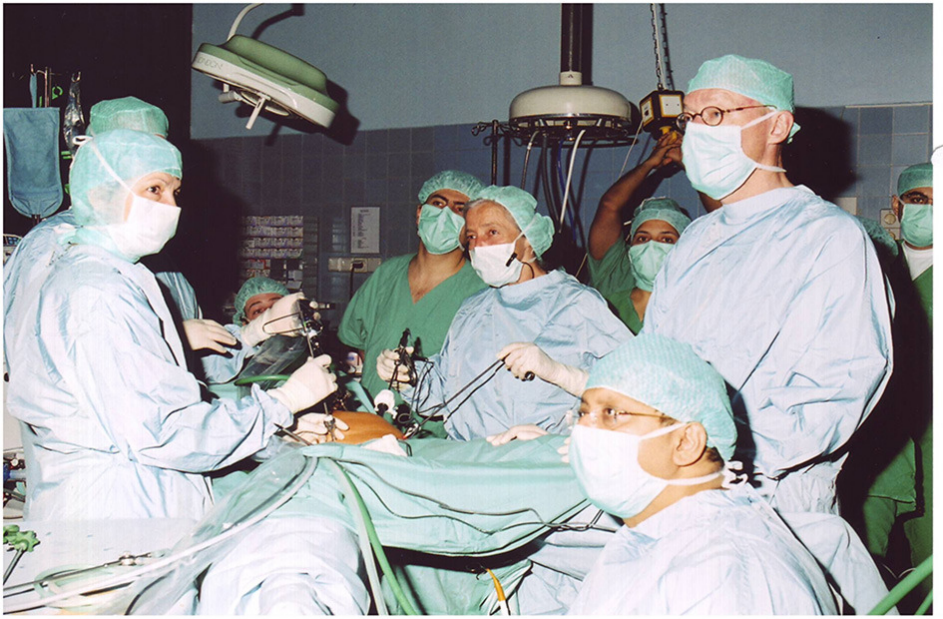
International laparoscopy course at the Endoscopy School of Kiel with Professor Liselotte Mettler. Source: Department of Obstetrics and Gynecology, University of Kiel.

Semm did not keep his knowledge to himself. Rather, he made it accessible to one and all. In his view, the art of laparoscopic surgery could not be learned by assisting an operation, as was the case for laparotomy. In 1985 he developed the Pelvi trainer, a phantom device for drilling operations (Figure 5) (40). The Kiel School of Gynecological Endoscopy was founded in 1990. Semm no longer needed to transport his technique elsewhere; he could make it accessible locally to one and all. A number of national and international courses have been held since this time (as shown in Figure 14).

### Excursus: Anatomical Illustration—Past and Present

**Figure 15:**
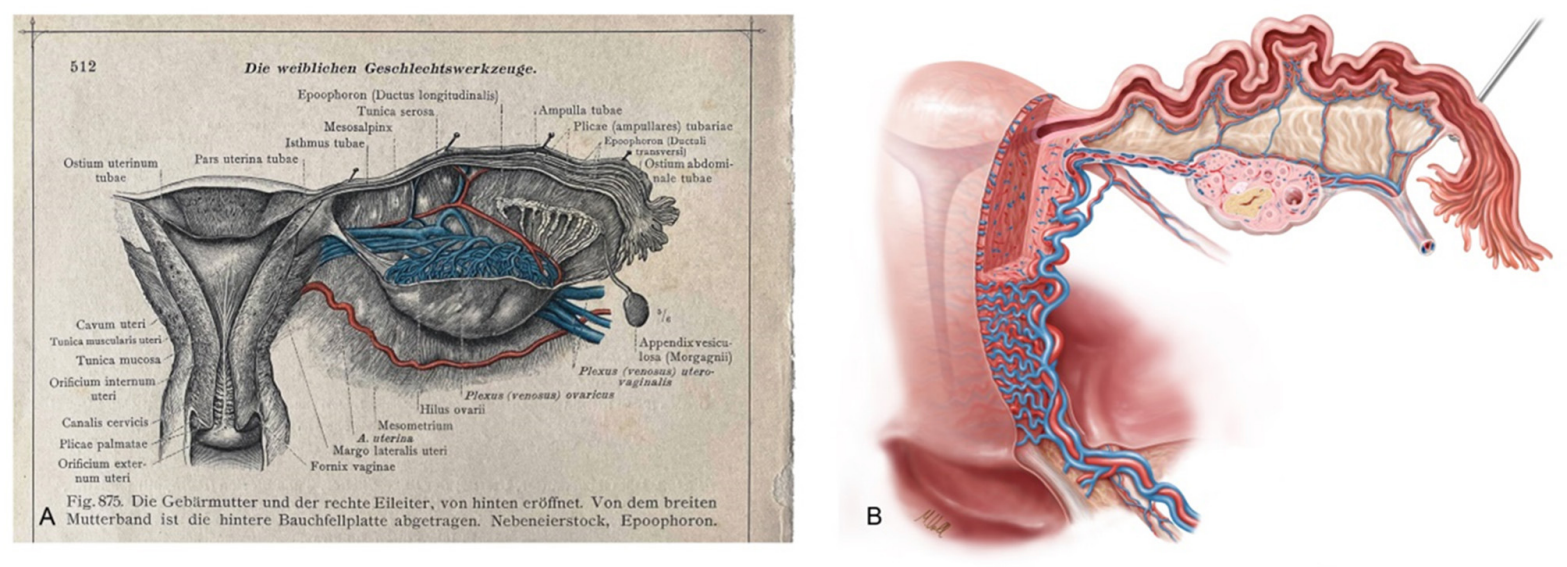
**(A)** Anatomical illustration of the uterus, adnexa and parametrium in an ancient anatomical atlas (Dr. Carl Toldt, Berlin and Wien 1903 [44]) and **(B)** modern female anatomical illustration generated by Markus Voll.

Viewing the transformations in surgery along with innovations in supporting medical specialties such as anesthesiology and intensive medicine, infectious diseases, pathology and others, it is almost disconcerting to note that illustrations of human anatomy, a basis for all medical specialties, were available in similar excellent quality 120 years ago and were by no means inferior to current visual presentations of human anatomy (as shown in Figure 15) (44).

### Modern Era of Laparoscopy

The introduction of endoscopy in surgical practice is one of the greatest success stories in the history of medicine. This historical overview shows that laparoscopy has developed at an incredible pace in the last two decades and created unprecedented opportunities in gynecology.

Current developments are mainly focused on improving image display and making it more realistic. Furthermore, we have experienced consistent progress in robot-assisted surgery and new surgical accesses such as natural orifice transluminal endoscopic surgery (NOTES) or access via a single trocar (single-port technique). Based on findings in pelvic neurofunctional anatomy and the introduction of laparoscopy in the dissection and visualization of the pelvic nerves Marc Possover introduced the concept of “Neuropelveology” in clinical practice. In 2014 the International Society of Neuropelveology was founded (45).

In an age of increasing specialization in medicine, the interdisciplinary approach is gaining importance and offers opportunities for endoscopic surgery (46). Thanks to the outstanding quality of digital imaging, operations can be shared more easily with surgeons of various specialties. This progress has been further aided by the development of new and user-friendly instruments that can be used in several specialties, and the simultaneous use of two consoles in robot-assisted surgery. Besides, modern operating rooms permit a rapid exchange of surgeons (47). Modern communication systems and telementoring allow the exchange of information over long distances. Thus, diverse specialties or surgeons can communicate intraoperatively without being physically present at the same site (48).

The disadvantages include the high cost of installation and maintenance in robot-assisted procedures, which usually involve long operating times at least at the beginning and are associated with a renewed learning curve even for experienced laparoscopists. Furthermore, doctors as well as nursing staff must be trained in the use of the robot system (49, 50). Docking and the placement of trocars, and the unfamiliar actions at the console take more time in the initial phase. Notwithstanding the existing disadvantages of robot-assisted surgery, technological developments in this field will lead to further dissemination of miniaturized and economical integrated systems in the foreseeable future even in gynecology, and further enlarge the spectrum of minimally invasive operation techniques (5, 51).

## Conclusion for Clinical Practice

We await the coming decades of development in medical technology with a tear in one eye and a smile in the other. With a smile because medicine and patient care will benefit from rapid global technological progress. With a tear because we do not know whether the original essence of our existence, namely exercising our manual skills on human beings, will be abolished in the near future.

## Author Contributions

IA, DF, UM, LM, JP, NM, MB, GG, and AL contributed equally to the formation, writing, and editing of this manuscript. All authors contributed to the article and approved the submitted version.

## Conflict of Interest

The authors declare that the research was conducted in the absence of any commercial or financial relationships that could be construed as a potential conflict of interest. The handling editor RW declared a past co-authorship with one of the author IA.

## Publisher's Note

All claims expressed in this article are solely those of the authors and do not necessarily represent those of their affiliated organizations, or those of the publisher, the editors and the reviewers. Any product that may be evaluated in this article, or claim that may be made by its manufacturer, is not guaranteed or endorsed by the publisher.

## References

[1] Alkatout I Holthaus B Wedel T Mettler L Ackermann J Maass N. Entwicklung der minimal-invasiven Chirurgie in der Gynäkologie und Überwindung assoziativer Herausforderungen. Der Gynäkologe. (2018) 51:737–43. 10.1007/s00129-018-4292-7

[2] Alkatout I. An atraumatic retractor for interdisciplinary use in conventional laparoscopy and robotic surgery. Minim Invasive Ther Allied Technol. (2018) 27:265–71. 10.1080/13645706.2018.1440244 29457928

[3] Alkatout I Mettler L. Hysterectomy a comprehensive surgical approach. J Turk Ger Gynecol Assoc. (2017) 18:221–3. 10.4274/jtgga.2017.0097 29278237 PMC5776163

[4] Semm K. Pelviskopie und Hysteroskopie. Farbatlas und Lehrbuch. Stuttgart: F.K. Schattauer Verlag GmbH (1976). p. 7–14.

[5] Schollmeyer T Semm K Schollmeyer M Mettler L. Practical Manual for Laparoscopic Hysteroscopic Gynecological Surgery. 2 Edn. In: Schollmeyer T Mettler L Rüther D. eds. New Delhi: Jaypee Brothers Medical Publishers (2013) p. 3–11. 10.5005/jp/books/11931_23

[6] Kelling G. Über Oesophagoskopie, Gastroskopie und Koelioskopie. Münchener Medizinische Wochenschrift (1902). p. 49:21.

[7] Hatzinger M Badawi JK Häcker A Langbein S Honeck P Alken P. [Georg Kelling (1866–1945): the man who introduced modern laparoscopy into medicine]. Urologe A. (2006) 45:868–71. 10.1007/s00120-006-1068-9 16773385

[8] Schollmeyer T Soyinka AS Schollmeyer M Meinhold-Heerlein I. Georg Kelling (1866–1945): the root of modern day minimal invasive surgery. A forgotten legend? Arch Gynecol Obstet. (2007) 276:505–9. 10.1007/s00404-007-0372-y 17458553

[9] Litynski GS. Kurt Semm and the fight against skepticism: endoscopic hemostasis, laparoscopic appendectomy, and Semm's impact on the “laparoscopic revolution”. JSLS. (1998) 2:309–13. 9876762 PMC3015306

[10] Semm K. Endoscopic appendectomy. Endoscopy. (1983) 15:59–64. 10.1055/s-2007-1021466 6221925

[11] Arnaud de Ronsil G. Memoires de chirurgie, avec quelques remarques historiques sur l'etat de la médecine et de la chirurgie en France et en Angleterre. Paris: Londres chez J. Nourse (1768).

[12] Bozzini P. Der Lichtleiter oder die Beschreibung einer einfachen Vorrichtung und Ihre Anwendung zur Erleuchtung innerer Höhlen und Zwischenräume des lebenden animalischen Körpers. Weimar: Verlag des Landes Industrie Comptoir (1807).

[13] Desormeaux AJ. De'Lendoscope et ses applications au diagnostic et au traitement des affections de l'urethre et de la vessie. Baillière (1865).

[14] Nitze M. Über die Behandlungsmethode der Höhlen des menschlichen Körpers. Wien Med Wschr. (1879) 24:851–8.

[15] Leiter J. Elektro-endoskopische Instrumente. Wien (1880).

[16] Mikulicz J. Gastroskopie und oesophagoskopie. Verh. dt. Ges. Chir. Berlin. (1882) 11:31–8.

[17] Litynski GS Paolucci V. Origin of laparoscopy: coincidence or surgical interdisciplinary thought? World J Surg. (1998) 22:899–902. 10.1007/s002689900490 9673567

[18] Litynski GS. Endoscopic surgery: the history, the pioneers. World J Surg. (1999) 23:745–53. 10.1007/s002689900576 10415199

[19] Hatzinger M Häcker A Langbein S Kwon S Hoang-Böhm J Alken P. Hans Christian Jacobaeus (1879–1937). Der Urologe. (2006) 45:1184–6. 10.1007/s00120-006-1069-8 16773384

[20] Kaiser AM Corman ML. History of laparoscopy. Surg Oncol Clin N Am. (2001) 10:483–92. 10.1016/S1055-3207(18)30045-0 11685923

[21] Morgenstern L. The first laparoscopist in the United States: Bertram m. Bernheim, MD. Surg Innov. (2007) 14:241–2. 10.1177/1553350607309433 18178910

[22] Goetze O. Die Röntgendiagnostik bei gasgefüllter Bauchhöhle; eine neue Methode. Munch Med Wochenschr. (1918) 65:1275–80.

[23] Korbsch R. Lehrbuch der Laparoskopie und Thorakoskopie. München: Lehmann (1927).

[24] Kalk H. Erfahrungen mit der Laparoskopie (zugleich mit Beschreibung eines neuen Instrumentes). Z Klin Med. (1929) 111:304–348.

[25] Fervers C. Die Laparoskopie mit dem Cystoscop. Ein Beitrag zur Vereinfachung der Technik und zur endoskopischen Strangdurchtrennung in der Bauchhöhle. Med Klin. (1933) 29:1042–5.

[26] Morgenstern L. No surgeon he. John C. Ruddock MD, F.A.C.P., pioneer in laparoscopy. Surg Endosc. (1996) 10:617–8. 10.1007/BF00188512 8662397

[27] Veress J. Neues Instrument zur Ausführung von Brust- oder Bauch Punktionen und Pneumothoraxbehandlung. Dtsch Med Wochenschr. (1938) 64:1480–1. 10.1055/s-0028-112340126606158

[28] Palmer R. La coelioscopie gynecologique. rapport du Prof. Mocquot. Acad De Chir. (1946) 72:363-8

[29] Decker A. Pelvic culdoscopy. In: Progress in Gynecology, Meigs JV editor. New York, NY: Grüne & Stratton (1946) p. 95–9.

[30] Frangenheim H. Die Laparoskopie und die Culdoskopie in der Gynäkologie. Stuttgart: Thieme Verlag (1959).

[31] Frangenheim H. Die Laparoskopie in der Gynäkologie, Chirurgie und Pädiatrie: Lehrbuch und Atlas. Stuttgart: Thieme Verlag (1970).

[32] Schollmeyer M Schollmeyer T. Georg Kelling und die sächsischen Wurzeln der Laparoskopie – 100 Jahre Laparoskopie. Siebenlehn: Verein Oschatzer Frauenärzte e.V., Druckerei Wagner, Verlag und Werbung GmbH (2001).

[33] Semm K. Operationen ohne Skalpell: Ein Gynäkologe als Wegbereiter der Minimal invasiven Medizin (Autobiographie). Landsberg: Ecomed Verlag (2002).

[34] Litynski GS. Erich Mühe and the rejection of laparoscopic cholecystectomy (1985): a surgeon ahead of his time. JSLS. (1998) 2:341–6. 10036125 PMC3015244

[35] Mühe E 296. Die erste Cholecystektomie durch das Laparoskop. Langenbecks Arch Chiv (1986) 369:804. 10.1007/BF01274615

[36] Reich H. Laparoscopic hysterectomy. Surg Laparosc Endosc. (1992) 2:85–8. 1341510

[37] Wickham JE. The new surgery. Br Med J. (1987) 295:1581–2. 10.1136/bmj.295.6613.1581 3121078 PMC1257475

[38] Schlich T Tang CL. Patient choice and the history of minimally invasive surgery. Lancet. (2016) 388:1369–70. 10.1016/S0140-6736(16)31738-X

[39] Frampton S Kneebone RL. John Wickham's New Surgery: ‘Minimally Invasive Therapy’, Innovation, and approaches to medical practice in twentieth-century Britain. Soc History Med. (2017) 30:544–66. 10.1093/shm/hkw074 29713119 PMC5914418

[40] Semm K. Pelvi-Trainer, ein Übungsgerät für die operative Pelviskopie zum Erlernen endoskopischer Ligatur und Nahttechniken [Pelvi-trainer, a training device in operative pelviscopy for teaching endoscopic ligation and suture technics]. Geburtshilfe Frauenheilkd. (1986) 46:60–2. 10.1055/s-2008-1036165 3514362

[41] Semm K. MIC. Bessere Lebensqualität durch Minimal Invasive Chirurgie (Better Quality of Life Through Minimally Invasive Surgery), 1 Edn. Kiel (1991). p. 18–42. 10.3109/13645709109152795

[42] Semm K. Gynäkologische Pelviskopie - Chromopertubation. Film D1100 des IWF Göttingen (1973).

[43] Semm K. Gynäkologische Pelviskopie - Verwachsungen. Film D1101 des IWF Göttingen (1973).

[44] Toldt C. Anatomischer Atlas. Für Studierende und Ärzte. Dritte, vermehrte und verbesserte Auflage, Vierte Lieferung. Berlin, Wien: Urban & Schwarzenberg Verlag (1903) p. 512.

[45] Possover M Forman A Rabischong B Lemos N Chiantera V. Neuropelveology: new groundbreaking discipline in medicine. J Minim Invasive Gynecol. (2015) 22:1140–1. 10.1016/j.jmig.2015.06.009 26099648

[46] Alkatout I Egberts JH Mettler L Doniec M Wedel T Jünemann KP. Interdisziplinäre Diagnostik und Therapie der tief infiltrierenden Endometriose [Interdisciplinary Diagnosis and Treatment of Deep Infiltrating Endometriosis]. Zentralbl Chir. (2016) 141:630–638. 10.1055/s-0034-1383272 25723864

[47] Hanly EJ Miller BE Kumar R Hasser CJ Coste-Maniere E Talamini MA. Mentoring console improves collaboration and teaching in surgical robotics. J Laparoendosc Adv Surg Tech A. (2006) 16:445–51. 10.1089/lap.2006.16.445 17004866

[48] Sebajang H Trudeau P Dougall A Hegge S McKinley C Anvari M. The role of telementoring and telerobotic assistance in the provision of laparoscopic colorectal surgery in rural areas. Surg Endosc. (2006) 20:1389–93. 10.1007/s00464-005-0260-0 16823656

[49] Schollmeyer T Mettler L Jonat W Alkatout I. Roboterchirurgie in der Gynäkologie. Der Gynäkologe. (2011) 44:196–201. 10.1007/s00129-010-2709-z

[50] Herron DM Marohn M The SAGES-MIRA Robotic Surgery Consensus Group. A consensus document on robotic surgery. Surg Endosc. (2008) 22:313–25; discussion 311-2. 10.1007/s00464-007-9727-5 18163170

[51] Schollmeyer T Elessawy M Chastamouratidhs B Alkatout I Meinhold-Heerlein I Mettler L. Hysterectomy trends over a 9-year period in an endoscopic teaching center. Int J Gynaecol Obstet. (2014) 126:45–9. 10.1016/j.ijgo.2013.12.017 24825496

[52] Alkatout I Pape JM Mechler U Mettler L Maass N Freytag D. Geschichte und Pioniere der Laparoskopie. Der Gynäkologe. (2021). 10.1007/s00129-021-04871-9

